# Proteomic Profiling Reveals the Molecular Changes of Insomnia Patients

**DOI:** 10.1155/2021/6685929

**Published:** 2021-01-13

**Authors:** Guanying Wang, Xiaojuan Ren, Xingping Zhang, Qingquan Wang, Tao Liu, Ning Deng, Deqi Yan

**Affiliations:** ^1^Xinjiang Medical University, Xinshi District, Urumqi 830011, China; ^2^Traditional Chinese Medicine Hospital Affiliated to Xinjiang Medical University, Saybagh District, Urumqi 830000, China; ^3^College of Traditional Chinese Medicine, Xinjiang Medical University, Xinshi District, Urumqi 830011, China; ^4^Postdoctoral Workstation of Traditional Chinese Medicine Hospital of Xinjiang Uygur Autonomous Region, Saybagh District, Urumqi 830000, China

## Abstract

**Background:**

Insomnia is an economic burden and public health problem. This study is aimed at exploring potential biological pathways and protein networks for insomnia characterized by wakefulness after sleep.

**Method:**

Proteomics analysis was performed in the insomnia group with wakefulness and the control group. The differentially expressed proteins (DEPs) were enriched; then, hub proteins were identified by protein-protein interaction (PPI) network and verified by parallel reaction monitoring (PRM).

**Results:**

Compared with the control group, the sleep time and efficiency of insomnia patients were decreased, and awakening time and numbers after sleep onset were significantly increased (*P* < 0.001). The results of proteomic sequencing found 68 DEPs in serum under 1.2-fold changed standard. These DEPs were significantly enriched in humoral immune response, complement and coagulation cascades, and cholesterol metabolism. Through the PPI network, we identified 10 proteins with the highest connectivity as hub proteins. Among them, the differential expression of 9 proteins was verified by PRM.

**Conclusion:**

We identified the hub proteins and molecular mechanisms of insomnia patients characterized by wakefulness after sleep. It provided potential molecular targets for the clinical diagnosis and treatment of these patients and indicated that the immune and metabolic systems may be closely related to insomnia characterized by wakefulness after sleep.

## 1. Introduction

Insomnia is defined as the difficulty in starting or maintaining sleep or nonrestorative sleep and the consequences of the day, such as fatigue, reduced attention, or daytime distress [1]. Insomnia is a common health problem and the second largest mental disorder [2]. About one-third of the general population has insomnia symptoms, and the estimated prevalence rate ranges from 10% (adults) to 22% (elderly) [3, 4]. A high incidence rate worldwide reflects the prevalence of insomnia [5]. Importantly, the effects of developing insomnia may not be limited to the long-term and reproducibility of insomnia. Insomnia is associated with cognitive impairment, decreased work efficiency, decreased quality of life, mental illness complications, higher medical costs, and higher risk of death [6, 7]. The mechanisms for the development and maintenance of insomnia are crucial for identifying treatment and prevention strategies to improve insomnia and its associated incidence rate.

The clinical features of insomnia include difficulty in falling asleep, difficulty in maintaining continuity of sleep (waking up in the middle of the night, difficulty in resuming sleep), or getting up earlier than required, regardless of sleep status (early morning insomnia) [8]. The latter two have the characteristics of easy to wake up after sleep and have the characteristics of less sleep time. Epidemiological studies have shown that short sleep time is associated with increased obesity, diabetes, hypertension, and mortality [9]. The neurobiological mechanism of insomnia may involve changes in brain regions related to cognition, emotion and sleep-wake, immune inflammation, and metabolism [10–12]. Molecular factors of sleep-wake regulation include sleep-promoting chemicals such as orexin, norepinephrine, and histamine [8]. Several studies have documented elevated metabolic parameters in patients with insomnia compared to those who sleep well [12, 13]. Sleep wakefulness promotes cerebral blood flow and glucose metabolism in the thalamus, hypothalamus, basal forebrain, basal ganglia, brainstem, and cerebellum [14]. Some studies believed that inflammation was a potential pathway through which insomnia and sleep deprivation could affect the risk of adult onset [15].

At present, there are few studies on the mechanism of insomnia in patients who are wakefulness after sleep. Here, we identified potential dysregulation mechanisms and biological targets by proteomics analysis of patient serum.

## 2. Materials and Methods

### 2.1. Participants

Participants were from a prospective survey conducted by the Traditional Chinese Medicine hospital affiliated to Xinjiang Medical University. Among the participants, 94 patients aged 20 to 50 years who met the criteria of wakefulness after sleep and 80 patients with good sleep were collected. The demographic, clinical characteristics, and sleep quality of the subjects were evaluated by effective research methods. To eliminate mental and medical disorders, all participants received questionnaires and interviews from certified clinicians and were assessed by nocturnal polysomnography (PSG) and Pittsburgh sleep quality index (PSQI). All experiments were carried out in accordance with The Code of Ethics of the World Medical Association. Research protocols were approved by the ethics committee and human use subcommittee of The First Affiliated Hospital of Xinjiang Medical University (Protocol No. 20120220-133). All participants signed written informed consent.

### 2.2. Sleep

Electroencephalogram (EEG), electrooculographic (EOG), and electromyographic (EMG) were recorded by PSG program. Participants sleep at their habitual bedtime. Participants who met the screening criteria completed PSG screening at home. PSG data were used to calculate the amount of sleep in each phase of the standard sleep structure variable, in minutes and percentages of total sleep time (TST). In addition, sleep latency (time from light off to the first stage of any sleep phase), number of awakenings, wake-time after sleep onset, rapid eye movement (REM) time, nonrapid eye movement (NREM) time, and sleep efficiency (SE; total sleep time divided by total recorded time) were also calculated.

The PSQI was used to assess sleep quality in the past month. Pittsburgh sleep diary was used to measure the daily sleep quality, fall asleep time, sleep time, sleep efficiency, sleep disorders, sleep drugs, and daytime dysfunction scores, and the total score of PSQI was calculated. Because each score was the result of subjective sleep complaints, the higher the score, the worse the sleep quality. A score greater than 10 has been recommended for the diagnosis of clinically significant insomnia.

### 2.3. Proteomic Detection

Serum samples of 9 patients of insomnia patients with wakefulness (D group) and 9 patients with good sleep (F group) were randomly selected. In the presence of protease inhibitors, the samples were lysed, and the total protein was extracted. For insomnia or control, every three samples were mixed into one group, and the fourth group was mixed with all samples. The protein concentration was determined by bicinchoninic acid (BCA) (Beyotime, Shanghai, China) kit, and the protein quality was detected by sodium dodecyl sulfate-polyacrylamide gel electrophoresis (SDS-PAGE).

The qualified protein was hydrolyzed by enzyme. After trypsin digestion, the peptide was desalted by Strata X C18 SPE column (Phenomenex) and vacuum-dried. Peptide was reconstituted in 0.5 M TEAB and processed according to the manufacturer's protocol for tandem mass tag (TMT) kit (Thermo, Bremen, Germany). Briefly, one unit of TMT reagent was thawed and reconstituted in acetonitrile. The peptide mixtures were then incubated for 2 h at room temperature and pooled, desalted, and dried by vacuum centrifugation. Nine samples from each group were randomly divided into three groups and a fourth group with all nine samples mixed. The bound peptides were eluted from the beads with 0.1% trifluoroacetic acid and combined and vacuum-dried. For liquid chromatograph-mass spectrometry/mass spectrometry (LC-MS/MS) analysis, the resulting peptides were desalted with C18 ZipTips (Millipore, Massachusetts, USA) according to the manufacturer's instructions.

The peptides were dissolved in 0.1% formic acid, directly loaded onto a reversed-phase analytical column. The peptides were subjected to NSI source followed by tandem mass spectrometry (MS/MS) in Q ExactiveTM Plus (Thermo, Bremen, Germany) coupled online to the UPLC. A data-dependent procedure that alternated between one MS scan followed by 20 MS/MS scans with 15.0 s dynamic exclusion. The resulting MS/MS data were processed using the MaxQuant search engine (v.1.5.2.8). Tandem mass spectra were searched against the SwissProt Human database concatenated with the reverse decoy database.

The multiple of protein expression between the insomnia group and control group was greater than or equal to 1.2, and *P* < 0.05, then differentially expressed proteins were obtained. Data analyzed in this study was submitted to the PRIDE database (accession number: PXD023246).

### 2.4. Enrichment Analysis

Gene Ontology (GO) of differentially expressed proteins was carried out by enrichGO functions of the clusterProfiler package. Kyoto Encyclopedia of Genes and Genomes (KEGG) pathway was obtained from ClueGo plug-in of Cytoscape software. Terms in GO and KEGG pathways were identified according to the cut-off criterion of *P* < 0.05. Gene set enrichment analysis (GSEA) software was used to identify the KEGG signaling pathway of differentially expressed proteins.

### 2.5. Protein Interaction

Based on the data of protein-protein interaction (PPI) in the string database, we constructed the PPI network of differentially expressed proteins. The PPI network was visualized using Cytoscape software through the combined score > 0.4 as the cut-off criterion.

### 2.6. Parallel Reaction Monitoring Data Acquisition

The serum samples of 9 patients with insomnia patients with wakefulness and 9 patients with good sleep were screened again to detect DEPs. The protein was extracted, and tryptic peptide is obtained according to the above method. After mixing the samples, the peptides were subjected to NSI source followed by tandem MS/MS in Q ExactiveTM Plus (Thermo, Bremen, Germany) coupled online to the UPLC. The predetermined parallel reaction monitoring (PRM) method was used for data collection. The resulting MS data were processed using Skyline (v.3.6).

### 2.7. Western Blot Analysis

For western blot analysis, we extracted proteins from whole blood samples of insomnia patients with wakefulness and healthy controls using a protein extraction kit (BestBio, Shanghai, China). Equal amounts of protein were separated using 10% SDS-PAGE and transferred to polyvinylidene difluoride (PVDF) membranes. The membrane containing the target protein was incubated with the primary antibody (Bioswamp, Wuhan, China) at 4°C for more than 10 hours, followed by another incubation with HRP-conjugated secondary antibodies (Bioswamp, Wuhan, China). Using GAPDH as the internal reference protein, the relative expression level of the protein was estimated by ImageJ.

### 2.8. Statistical Analysis

SPSS19.0 (IBM, NY, USA) for windows was used for statistical analysis. The continuous variable is mean ± standard deviation (SD). The demographic and sleep characteristics of each group were compared and analyzed by Student *t*-test or chi-square test. Statistical tests showed that *P* < 0.05 was significant.

## 3. Results

### 3.1. Sleep Characteristics

**Figure 1:**
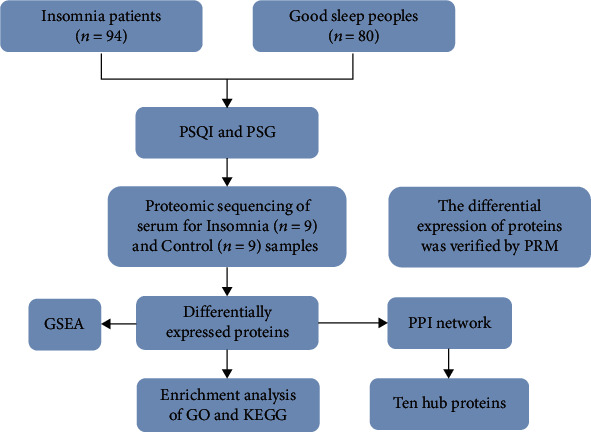
Flowchart of study.

The flowchart of this study is shown in Figure 1. The sociodemographic and PSG characteristics of the samples were shown in Table 1. In short, insomniacs were young to middle-aged adults (mean = 40.15 ± 6.601 y), with 33.0% of males. There was no significant difference in age and gender between groups. Compared with those with good sleep, insomnia patients were significantly worse in sleep quality and sleep time (PSQI, sleep efficiency, awakening time, and numbers) (*P* < 0.001).

### 3.2. Differentially Expressed Proteins in Insomnia Patients with Wakefulness

**Figure 2:**
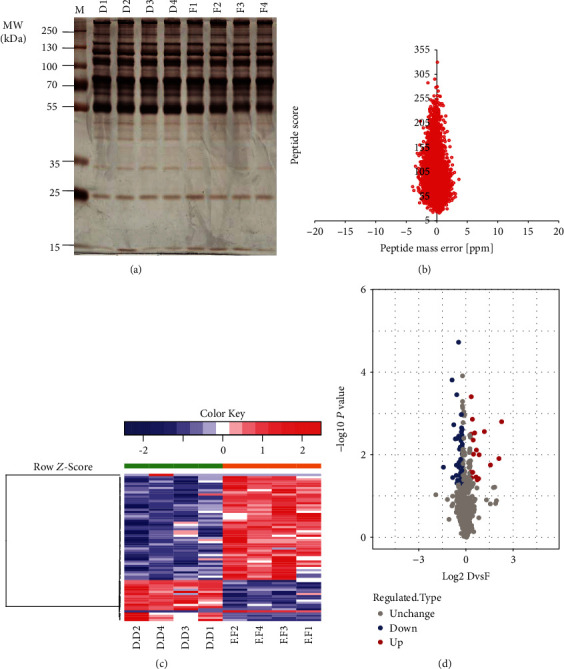
Calculation of differentially expressed proteins. (a) The protein integrity of 8 groups for mixed samples was detected by SDS-PAGE. (b) The error distribution between the true and theoretical values of the relative molecular weights of all matched peptides. (c) The differential expression protein between insomnia and control group was calculated with a 1.2-fold change standard. (d). Volcano map of upregulated or downregulated differentially expressed proteins.

To screen for potential differences in protein expression between insomnia patients and controls, TMT proteomics of serum were performed. After protein extraction, 8 groups of mixed samples (4 groups of insomnia patients and 4 groups of controls) were evaluated by SDS-PAGE, which showed reliable protein integrity (Figure 2(a)). After TMT labeling, all samples were collected for peptide separation and identification. A total of 275653 MS/MS spectra were obtained, of which 16595 were matched spectra. Then, 676 proteins were extracted from 4305 unique peptides. The center axis of the peptide mass axis is within 1 ppm, and the main body is within ±5 ppm, indicating that the mass axis of mass spectrometry is accurate and stable (Figure 2(b)).

To explore the potential changes of proteomic profiles between insomnia patients and normal controls, 68 differentially expressed proteins were identified with a 1.2-fold change criterion (*P* < 0.05) (Figure 2(c), Table S1). There were 18 DEPs with a 1.2-fold increase and 50 DEPs with a 1.2-fold decrease (Figure 2(d)).

### 3.3. Biological Function and Molecular Pathways of Insomnia with Wakefulness

**Figure 3:**
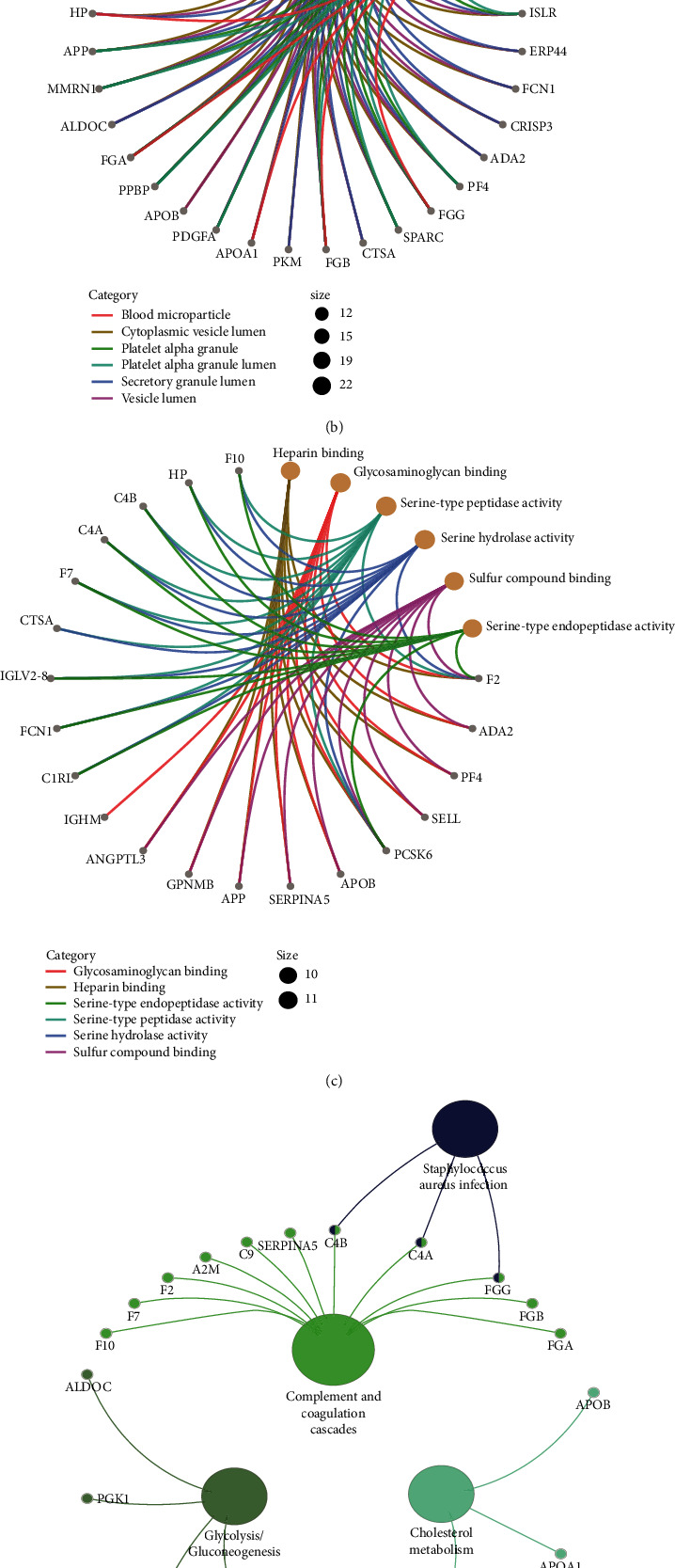
GO function and KEGG pathway of differentially expressed proteins. (a) Differentially expressed proteins were involved in biological processes. (b) Differentially expressed proteins were involved in cell composition. (c) Differentially expressed proteins were involved in molecular function. The top 6 terms with the most significant *P* value. (d) Differentially expressed proteins were significantly involved in KEGG pathways. (e) The KEGG results of GSEA. D: insomnia group; F: control group.

In the results of biological process (BP) in enrichment, the differentially expressed proteins are mainly involved in humoral immune response, complement activation, etc. (Figure 3(a)). In cell components (CC), the differentially expressed proteins were significantly involved in cytoplasmic vesicle lumen, vascular lumen, etc. (Figure 3(b)). In the process of molecular function (MF), the differentially expressed proteins are mainly involved in heparin binding, glycosaminoglycan binding, etc. (Figure 3(c)). In addition, we identified the KEGG signaling pathways including complement and coagulation cascades, cholesterol metabolism, glycolysis/gluconeogenesis, and staphylococcus aureus infection (Figure 3(d)). GSEA results showed that the insomnia characteristics of wakefulness related proteins were significantly clustered in the complement and coagulation cascades (Figure 3(e)).

### 3.4. Interaction Network of Differentially Expressed Proteins

**Figure 4:**
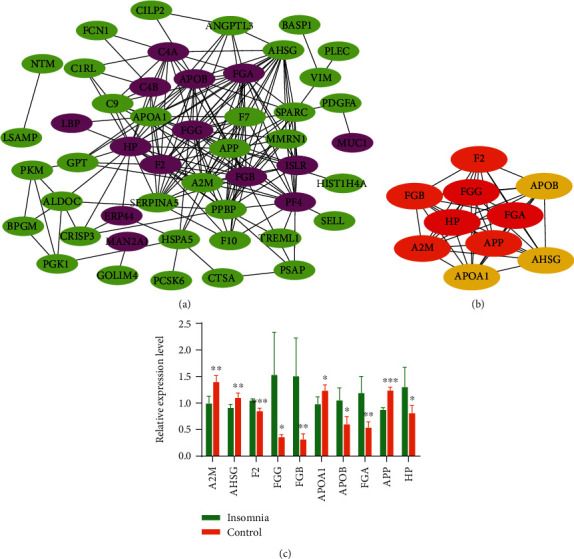
Screening and validation of hub proteins in PPI network. (a) PPI network of differentially expressed proteins. Pink nodes were upregulated proteins, and green nodes were downregulated proteins. (b) The top 10 proteins with the highest connectivity in the PPI network. (c) The differential expression of the top 10 proteins in the insomnia and control group. ^∗^*P* < 0.05, ^∗∗^*P* < 0.01, and ^∗∗∗^*P* < 0.001.

**Figure 5:**
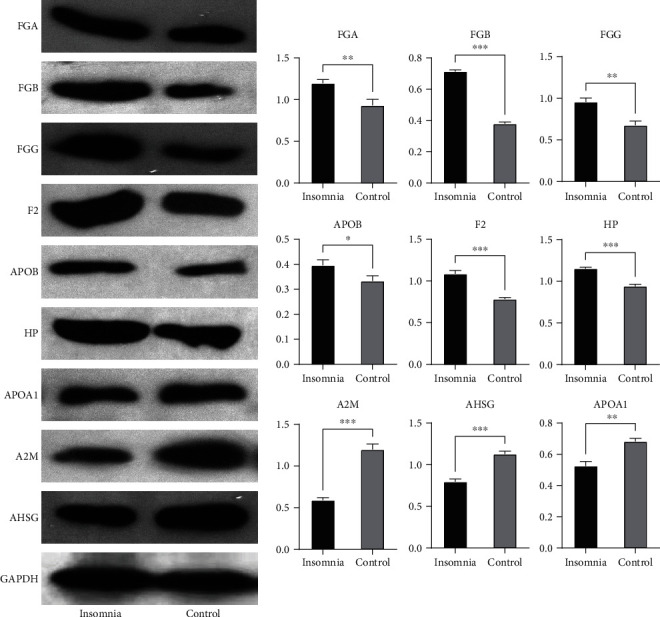
The expression of hub proteins in the insomnia and control group was detected by Western blot.

String is a database for predicting protein binding, which is used to predict protein interactions among identified differentially expressed proteins. The DEPs were mapped to the String website to obtain their PPI data. A PPI network with 49 nodes was obtained with a comprehensive score of ≥0.4 (Figure 4(a)). The 10 proteins with the highest connectivity with other nodes were selected as the hub proteins of the PPI network, suggesting that they may be closely related to insomnia (Figure 4(b)). Among them, the expression of F2, HP, FGA, FGB, FGG, and ApoB were upregulated in insomnia patients with wakefulness, and A2M, AHSG, APP, and ApoA1 were downregulated (Figure 4(c)). Importantly, through PRM analysis of 68 DEPs, 26 differentially expressed proteins were identified, including 9 hub proteins (Table 2). On the other hand, through Western blot experiments, we verified that the expression of FGG, FGB, FGA, APOB, F2, and HP was upregulated in insomnia patients with wakefulness compared with the control, while the expression of A2M, AHSG, and APOA1 was downregulated (Figure 5). A2M, F2, FGA, FGB, and FGG were significantly involved in the complement and coagulation cascades, while ApoA1 and ApoB were significantly involved in cholesterol metabolism.

## 4. Discussion

The medical field of sleep disorders attempts to define the subgroup of insomnia according to the etiology, age of onset, and the differences between objective and subjective sleep outcomes [16]. Through the objective clinical observation records of insomnia patients, this study found the insomnia subgroup characterized by wakefulness after sleep. Proteomics was used to identify the difference of serum protein expression in patients with this subtype, and related biological functions and signal pathways were identified. The hub proteins were screened by bioinformatics methods, which may be used as biomarkers and therapeutic targets for insomnia patients characterized by wakefulness after sleep.

This study found that patients with the characteristics of wakefulness after sleep generally have short sleep time and sleep fragmentation. In fact, this characteristic of insomnia has been confirmed by other studies [17, 18]. The wakefulness after sleep seriously affected the sleep time and sleep quality of patients. Sleep duration defined by PSG was short, which was associated with increased mortality [19]. Studies had shown that sleep restriction and disruption had adverse effects on appetite-regulating hormones, insulin sensitivity, systemic inflammatory markers, and autonomic nerve regulation and function [20–22].

Enrichment analysis showed that humoral immune response, complement and coagulation cascades, and cholesterol metabolism were the main molecular mechanisms related to DEPs. Sleep-immune interaction is a well-known phenomenon. By activating the immune system and releasing cytokines such as tumor necrosis factor (TNF) and interleukin-1- (IL-) 1 *β*, sleep regulation can be promoted [23]. In humoral signal transmission, peripheral cytokines can be transmitted to the brain central nervous system through a variety of ways [24]. These neurons express cytokine receptors that, once activated, send nerve signals to the brain. These cytokines activate microglia, which transmit this signal, leading to changes in behavior and sleep [25, 26]. A study found that during the whole night of sleep deprivation, the levels of IgG, IgM, and IgA in the morning cycle of human beings were increased, and the complement factors C3 and C5 were also increased [27]. The complement system is involved in the humoral immune response, and its improper activation plays a harmful role in diseases [28]. Animal studies showed that after 96 hours of REM sleep deprivation, the level of complement C3 increased temporarily [29]. Coagulation and immune response are closely related and interdependent processes. A study of middle-aged women with multiethnic groups suggested that the procoagulant process may be an important way of sleep disorders [30]. Improving the balance of glucose and cholesterol not only helped to control weight but also improved brain function and mood, which was essential for sleep [31]. A population-based study reported the association between insomnia and metabolic syndrome by comparing cholesterol metabolism [32]. Abnormal cholesterol metabolism may be associated with a higher risk of cardiovascular disease associated with poor sleep quality [33].

In the PPI network composed of DEPs, we identified 10 proteins with the highest degree of connectivity, and the differential expression of 9 proteins was verified by PRM. Among them, FGA, FGB, and FGG belong to fibrinogen, and the more severe insomnia symptoms are related to higher levels of fibrinogen [34]. Strengthening the balance of fibrinogen may promote the recovery of regulatory systems, thus, improving sleep fragmentation [35]. The results showed that the protein expression of ApoA 1 decreased in insomnia patients, while the expression of ApoB increased. Studies had shown that the increase of ApoA can improve insomnia symptoms [36]. Short sleep time was significantly associated with increased ApoB levels in women [37]. ApoA was a metabolic marker of high-density lipoprotein cholesterol (HDL-C). Increasing HDL-C level by 1 mg/dl can reduce the risk of cardiovascular disease by 2% to 3% [38]. *α* 2-Heremans Schmid glycoprotein (AHSG) is involved in important cellular physiological functions, such as cellular protein and fatty acid metabolism, and regulation of the acute inflammatory response [39]. AHSG, A2M, F2, and HP have not been reported to be associated with insomnia, but our study showed that they are significantly associated with insomnia. This needs further investigation by follow-up experiments.

Although this study provided new data for understanding the etiology of insomnia patients characterized by wakefulness after sleep, there were still some limitations. First, a part of DEGs identified by proteomics was verified by PRM, and these proteins may need to be replicated in other samples. Second, the current research does not explore the relationship between hub protein and insomnia characteristics. To sum up, through the comprehensive analysis of proteomics, enrichment, and PPI network data of insomnia patients, the current results showed that the disorder proteins involved in the immune and metabolic systems may be related to the pathogenesis of sleep characterized by wakefulness after sleep. The changes in immune and metabolic systems may be the results or the causes of insomnia, which need more research.

## 5. Conclusion

Compared with the good sleep group, the sleep quality and sleep time of insomnia patients characterized by wakefulness after sleep were significantly worse. The differentially expressed proteins are mainly involved in humoral immunity and cholesterol metabolism-related biological functions and signaling pathways. The hub proteins may be biomarkers and therapeutic targets for patients with insomnia.

## Figures and Tables

**Table 1:** The comparative characteristics of primary insomnia patients and sleep well control group.

Characteristic	Insomnia (94)	Control (80)	*t*/*χ*^2^	*P*
Gender (M)	31 (33.0%)	26 (32.5%)	*χ* ^2^ = 0.004	0.947
Age (years)	40.15 ± 6.601	40.43 ± 7.425	*t* = 0.259	0.795
Body mass index	23.54 ± 2.989	23.81 ± 3.954	*t* = 0.510	0.611
PSQI	13.64 ± 2.381	4.83 ± 1.391	*t* = 29.130	<0.001
TST (min)	412.82 ± 58.178	446.49 ± 41.504	*t* = 4.323	<0.001
Time in bed, min	520.66 ± 65.468	488.76 ± 38.437	*t* = 3.831	<0.001
Sleep efficiency (%)	79.67 ± 9.085	91.32 ± 3.906	*t* = 10.660	<0.001
Sleep onset latency, min	19.2 ± 18.456	9.23 ± 12.053	*t* = 4.138	<0.001
REM sleep, min	63.37 ± 32.213	68.52 ± 36.036	*t* = 0.996	0.321
REM%	15.29 ± 7.462	15.29 ± 7.869	*t* = 0.005	0.996
NREM sleep, min	350.34 ± 56.899	374.89 ± 57.704	*t* = 2.818	<0.01
NREM%	84.93 ± 7.512	84.27 ± 11.969	*t* = 0.439	0.661
Wake-time after sleep onset, min	75.24 ± 43.523	29.41 ± 18.433	*t* = 8.769	<0.001
Number of awakenings	13.98 ± 6.694	4.19 ± 2.591	*t* = 12.330	<0.001
Awakening time in TST (%)	18.99 ± 12.753	6.75 ± 4.369	*t* = 8.184	<0.001
Stage 1, min	44.60 ± 25.279	69.50 ± 64.836	*t* = 3.431	<0.001
Stage 1, %	11.15 ± 7.005	15.81 ± 15.072	*t* = 2.677	<0.01
Stage 2, min	285.21 ± 63.053	257.45 ± 81.882	*t* = 2.523	<0.05
Stage 2, %	68.75 ± 9.859	57.42 ± 17.093	*t* = 5.452	<0.001
Stage 3, min	20.53 ± 22.722	47.94 ± 29.182	*t* = 6.959	<0.001
Stage 3, %	5.07 ± 5.452	10.80 ± 6.627	*t* = 6.255	<0.001

PSQI: Pittsburgh Sleep Quality Index; TST: total sleep time; REM: rapid eye movement sleep; NREM: Nonrapid eye movement sleep.

**Table 2:** The results of PRM. D: insomnia group; F: control group.

Protein accession	Protein gene	Insomnia relative abundance	Control relative abundance	Insomnia/control ratio	Insomnia/control ratio (TMT)
P02679	FGG	2.00	0.00	∞	4.17
P01023	A2M	0.74	1.26	0.59	0.71
P02765	AHSG	0.91	1.09	0.84	0.83
P00734	F2	1.56	0.44	3.51	1.23
P04114	APOB	1.17	0.83	1.42	1.74
P02671	FGA	0.86	1.14	0.75	2.20
P02675	FGB	1.99	0.01	175.61	4.69
P02647	APOA1	0.91	1.09	0.83	0.80
P00738	HP	1.37	0.63	2.18	1.61

## Data Availability

The data is available in Table S1.

## Supplementary Materials

Supplementary Materials Table S1: differentially expressed proteins.
