# Supplementary material for: Proteomic Profiling Reveals the Molecular Changes of Insomnia Patients
Source: Biomed Res Int. 2021 Jan 13;2021:6685929. doi: 10.1155/2021/6685929 (PMC7822674; doi:10.1155/2021/6685929)
Supplement: Supplementary Materials — Table S1: differentially expressed proteins. [file 6685929.f1.pdf]

**Table S1. Differentially expressed proteins.**

| Accession | Protein description | RatioD/F | P valu | Gene name | D.D2 | D.D4 | D.D3 | D.D1 | F.F2 | F.F4 | F.F3 | F.F1 |
|---|---|---|---|---|---|---|---|---|---|---|---|---|
| Q16706 | Alpha-mannosidase 2 OS | 1.21 | 0.027 | MAN2A1 | 1.15 | 1.04 | 0.91 | 1.1 | 0.77 | 0.91 | 0.91 | 0.886 |
| P80723 | Brain acid soluble protei | 0.758 | 0.007 | BASP1 | 0.87 | 0.96 | 1.06 | 0.83 | 1.3 | 1.23 | 1.08 | 1.297 |
| Q13449 | Limbic system-associatec | 0.826 | 0.042 | LSAMP | 0.82 | 0.95 | 1.01 | 1.14 | 1.31 | 1.15 | 1.14 | 1.143 |
| P01023 | Alpha-2-macroglobulin C | 0.712 | 0.004 | A2M | 0.87 | 0.95 | 1 | 1.18 | 1.31 | 1.53 | 1.32 | 1.466 |
| P20815 | Cytochrome P450 3A5 O | 0.66 | 0.004 | CYP3A5 | 0.71 | | 0.73 | 0.64 | 1.1 | | 1.12 | 0.93 |
| P02748 | Complement component | 0.828 | 0.01 | C9 | 0.89 | 1.04 | 0.87 | 1.05 | 1.21 | 1.16 | 1.14 | 1.137 |
| Q9NZP8 | Complement C1r subcom | 0.811 | 0.004 | C1RL | 0.86 | 0.93 | 0.99 | 1.02 | 1.14 | 1.13 | 1.27 | 1.137 |
| P02765 | Alpha-2-HS-glycoprotein | 0.828 | 0.008 | AHSG | 0.96 | 0.9 | 0.85 | 0.97 | 1.01 | 1.08 | 1.15 | 1.192 |
| Q9HAT2 | Sialate O-acetylesterase ( | 0.821 | 0.018 | SIAE | 0.84 | 0.86 | 0.79 | | 1.01 | 0.93 | 1.09 | |
| O14498 | Immunoglobulin superfar | 1.219 | 0.003 | ISLR | 1.06 | 1.12 | 1.08 | 1.12 | 0.9 | 0.84 | 0.85 | 0.998 |
| Q9BS26 | Endoplasmic reticulum re | 1.432 | 0.003 | ERP44 | 1.18 | 1.11 | 1.25 | 1.07 | 0.73 | 0.77 | 0.75 | 0.976 |
| O00602 | Ficolin-1 OS=Homo sapi | 0.714 | 0.02 | FCN1 | 0.72 | 0.96 | 1.13 | 0.79 | 1.42 | 1.24 | 1.26 | 1.132 |
| P11021 | Endoplasmic reticulum cl | 0.833 | 0.006 | HSPA5 | 0.95 | 0.98 | 0.91 | 1.04 | 1.25 | 1.15 | 1.18 | 1.072 |
| P54108 | Cysteine-rich secretory p | 0.786 | 0.005 | CRISP3 | 0.83 | 0.93 | 1.01 | 1 | 1.28 | 1.13 | 1.24 | 1.132 |
| Q9P121 | Neurotrimin OS=Homo s | 0.796 | 0.041 | NTM | | | 0.95 | 0.97 | | | 1.26 | 1.145 |
| P00734 | Prothrombin OS=Homo s | 1.234 | 4E-04 | F2 | 1.03 | 1.06 | 1.06 | 1.08 | 0.92 | 0.83 | 0.87 | 0.805 |
| Q92484 | Acid sphingomyelinase-li | 0.781 | 0.022 | SMPDL3A | 0.91 | 0.87 | | 0.85 | 1 | 1.12 | | 1.245 |
| P14209 | CD99 antigen OS=Homo | 0.798 | 0.003 | CD99 | 0.87 | | 0.92 | 0.84 | 1.1 | | 1.14 | 1.053 |
| P01871 | Immunoglobulin heavy c | 0.819 | 0.039 | IGHM | 0.72 | 0.65 | 0.72 | 0.69 | 0.95 | 0.83 | 0.92 | 0.695 |
| P01709 | Immunoglobulin lambda | 0.753 | 0.033 | IGLV2-8 | | 0.73 | 0.66 | 0.78 | | 0.98 | 1.07 | 0.833 |
| P00558 | Phosphoglycerate kinase | 0.721 | 0.016 | PGK1 | 0.72 | 0.8 | 0.84 | 1.04 | 1.4 | 1.08 | 1.1 | 1.125 |
| Q9NZK5 | Adenosine deaminase 2 ( | 0.805 | 0.002 | ADA2 | 0.94 | 0.98 | 0.89 | 0.88 | 1.27 | 1.13 | 1.11 | 1.079 |
| Q96RT7 | Gamma-tubulin complex | 1.549 | 0.008 | TUBGCP6 | | 1.17 | | 1.1 | | 0.75 | | 0.718 |
| Q86VW0 | SEC14 domain and spect | 0.53 | 2E-04 | SESTD1 | 0.57 | 0.8 | 0.66 | 0.64 | 1.29 | 1.25 | 1.33 | 1.159 |
| P08670 | Vimentin OS=Homo sapi | 0.362 | 0.02 | VIM | 0.64 | | | 0.82 | 2.19 | | | 1.845 |
| P02776 | Platelet factor 4 OS=Hon | 2.877 | 0.018 | PF4 | 2.13 | | 1.01 | 1.15 | 0.58 | | 0.38 | 0.53 |
| P14151 | L-selectin OS=Homo sap | 0.764 | 0.007 | SELL | 0.86 | 0.96 | 0.98 | 1.07 | 1.45 | 1.24 | 1.25 | 1.143 |
| Q15149 | Plectin OS=Homo sapien | 0.545 | 0.036 | PLEC | 0.57 | | | 0.7 | 1.23 | | | 1.101 |
| O60832 | H/ACA ribonucleoprotei | 1.252 | 0.037 | DKC1 | | 0.89 | | 0.95 | | 0.76 | | 0.715 |
| P07602 | Prosaposin OS=Homo sa | 0.831 | 0.019 | PSAP | 0.79 | 0.86 | 1.02 | 0.98 | 1.08 | 1.09 | 1.15 | 1.077 |
| O00461 | Golgi integral membrane | 0.77 | 0.028 | GOLIM4 | 0.99 | | 0.9 | | 1.23 | | 1.22 | |
| P15941 | Mucin-1 OS=Homo sapie | 1.36 | 0.01 | MUC1 | | 1.15 | | 1.21 | | 0.85 | | 0.878 |
| Q86YW5 | Trem-like transcript 1 pro | 0.826 | 0.007 | TREML1 | 0.86 | 0.8 | 0.73 | 0.87 | 0.98 | 0.97 | 1.06 | 0.937 |
| P02679 | Fibrinogen gamma chain | 4.174 | 0.013 | FGG | 2.65 | 1.06 | 0.88 | 1.56 | 0.42 | 0.36 | 0.35 | 0.341 |
| P09486 | SPARC OS=Homo sapie | 0.821 | 0.003 | SPARC | 0.98 | 0.9 | 0.9 | 0.93 | 1.08 | 1.11 | 1.25 | 1.069 |
| P10619 | Lysosomal protective pro | 0.64 | 0.032 | CTSA | | 0.83 | | 0.93 | | 1.3 | | 1.458 |
| P02675 | Fibrinogen beta chain OS | 4.694 | 0.002 | FGB | 2.48 | 1.09 | 0.88 | 1.6 | 0.41 | 0.23 | 0.41 | 0.249 |
| P13489 | Ribonuclease inhibitor O | 0.806 | 0.037 | RNH1 | | 0.92 | 1.01 | 1.05 | | 1.18 | 1.37 | 1.14 |
| A0A075B | Immunoglobulin lambda | 0.613 | 0.004 | IGLV3-9 | 0.65 | 0.53 | 0.77 | 0.61 | 1.07 | 1.07 | 1.2 | 0.833 |
| P14618 | Pyruvate kinase PKM OS | 0.829 | 0.002 | PKM | 0.96 | 0.96 | 0.9 | 1 | 1.16 | 1.07 | 1.23 | 1.149 |
| P02647 | Apolipoprotein A-I OS=I | 0.797 | 0.024 | APOA1 | 0.84 | 0.99 | 1.15 | 0.97 | 1.35 | 1.23 | 1.27 | 1.111 |
| P29122 | Proprotein convertase sul | 0.829 | 0.02 | PCSK6 | 0.87 | 0.89 | 0.94 | | 1.06 | 1.17 | 1.01 | |
| Q8IUL8 | Cartilage intermediate lay | 0.652 | 4E-04 | CILP2 | 1.03 | 0.93 | 0.96 | 0.85 | 1.57 | 1.33 | 1.54 | 1.345 |
| Q9H299 | SH3 domain-binding glut | 0.658 | 0.049 | SH3BGRL3 | 0.68 | 0.58 | 0.69 | 0.86 | 1.25 | 0.97 | 1.33 | 0.714 |
| P04085 | Platelet-derived growth fa | 0.571 | 0.002 | PDGFA | 0.68 | 0.88 | 0.78 | 0.93 | 1.53 | 1.37 | 1.67 | 1.156 |
| P18428 | Lipopolysaccharide-bindi | 1.703 | 0.038 | LBP | | 1.18 | 1.36 | 0.95 | | 0.76 | 0.5 | 0.786 |
| O76074 | cGMP-specific 3',5'-cycli | 1.528 | 0.035 | PDE5A | 1.1 | | 1.19 | | 0.7 | | 0.8 | |
| Q8N3X6 | Ligand-dependent nuclea | 0.772 | 0.017 | LCORL | | 0.96 | 0.84 | 1.03 | | 1.18 | 1.3 | 1.184 |
| P22304 | Iduronate 2-sulfatase OS= | 0.823 | 0.047 | IDS | 0.84 | 0.9 | 1.05 | | 1.18 | 1.11 | 1.1 | |
| P0DOY3 | Immunoglobulin lambda | 0.774 | 0.013 | IGLC3 | 0.75 | 0.76 | 0.91 | 0.85 | 1.13 | 1.06 | 1.15 | 0.895 |
| P04114 | Apolipoprotein B-100 OS | 1.744 | 0.01 | APOB | 1.35 | 1.11 | 0.83 | 0.95 | 0.47 | 0.8 | 0.59 | 0.566 |

| | | | | | | | | | | | | |
|---|---|---|---|---|---|---|---|---|---|---|---|---|
| Q9ULV0 | Unconventional myosin-V | 0.727 | 0.043 | MYO5B | | 0.8 | 0.77 | | | 1.01 | 1.15 | |
| P02775 | Platelet basic protein OS= | 0.735 | 0.007 | PPBP | 0.81 | 0.9 | 0.84 | 1.03 | 1.18 | 1.17 | 1.43 | 1.088 |
| P02671 | Fibrinogen alpha chain O | 2.197 | 0.003 | FGA | 1.51 | 1.01 | 0.87 | 1.4 | 0.7 | 0.53 | 0.48 | 0.474 |
| P09972 | Fructose-bisphosphate ald | 0.776 | 0.031 | ALDOC | 0.95 | | 0.87 | | 1.19 | | 1.16 | |
| P08709 | Coagulation factor VII O | 0.757 | 0.018 | F7 | 0.75 | 0.87 | | 0.92 | 1.04 | 1.14 | | 1.179 |
| P0C0L4 | Complement C4-A OS=H | 1.342 | 0.004 | C4A | 1.24 | 1.11 | 1.42 | 1.12 | 0.96 | 0.93 | 0.84 | 0.918 |
| P0C0L5 | Complement C4-B OS=H | 1.298 | 0.001 | C4B | 1.21 | 1.06 | 1 | 1.14 | 0.86 | 0.86 | 0.88 | 0.799 |
| Q13201 | Multimerin-1 OS=Homo | 0.796 | 0.001 | MMRN1 | 0.87 | 0.92 | 0.92 | 1.01 | 1.22 | 1.19 | 1.17 | 1.093 |
| P05154 | Plasma serine protease in | 0.81 | 0.049 | SERPINA5 | 0.88 | 0.89 | 0.8 | 1.09 | 1.14 | 1.15 | 1.08 | 1.15 |
| P05067 | Amyloid-beta precursor p | 0.707 | 2E-05 | APP | 0.85 | 0.88 | 0.87 | 0.92 | 1.21 | 1.19 | 1.27 | 1.303 |
| P00738 | Haptoglobin OS=Homo s | 1.605 | 0.041 | HP | 1.42 | 1.29 | 1.7 | 0.84 | 0.87 | 0.83 | 0.95 | 0.628 |
| P00742 | Coagulation factor X OS= | 0.812 | 0.024 | F10 | 0.93 | 0.91 | 0.92 | 0.95 | 1.02 | 1.09 | 1.22 | 1.252 |
| P24298 | Alanine aminotransferase | 0.808 | 0.018 | GPT | 0.92 | 0.92 | | | 1.17 | 1.11 | | |
| Q14956 | Transmembrane glycopro | 1.316 | 0.027 | GPNMB | 1.32 | 1.18 | 1.02 | 1.32 | 0.81 | 0.93 | 0.83 | 1.114 |
| P62805 | Histone H4 OS=Homo sa | 0.642 | 0.018 | HIST1H4A | 0.93 | 0.68 | 0.73 | 0.74 | 1.49 | 1.18 | 0.86 | 1.254 |
| Q9Y5C1 | Angiopoietin-related prot | 0.817 | 0.019 | ANGPTL3 | 0.74 | 0.88 | 0.9 | 0.92 | 1.15 | 1.04 | 1.08 | 0.944 |
| P07738 | Bisphosphoglycerate mut | 0.677 | 0.042 | BPGM | 0.6 | 0.93 | 0.75 | 1.13 | 1.22 | 1.17 | 1.57 | 1.067 |
